# Understanding the psychosocial impact of the COVID-19 pandemic on Latinx emerging adults

**DOI:** 10.3389/fpsyg.2023.1066513

**Published:** 2023-02-20

**Authors:** Natalia Jaramillo, Erika D. Felix

**Affiliations:** ^1^Department of Psychiatry and Biobehavioral Sciences, University of California, Los Angeles, Los Angeles, CA, United States; ^2^Department of Counseling, Clinical, and School Psychology, University of California, Santa Barbara, Santa Barbara, CA, United States

**Keywords:** Latinx, emerging adult, COVID-19, psychosocial, adjustment

## Abstract

There is growing evidence about the potential negative psychosocial impact of the COVID-19 pandemic on ethnoracially minoritized young adults. Emerging adulthood is a developmental stage (ages 18-29 years old) that is characterized by identity exploration, instability, self-focus, feeling “in between” and having a sense of possibilities. Latinx emerging adults have reported significant socio-emotional consequences as a result of the COVID-19 pandemic. The aim of this study was to explore the psychosocial impact of the COVID-19 pandemic on Latinx emerging adults (N = 31; ages 18-29) in California and Florida through online focus group interviews. A qualitative constructivist grounded theory approach was used in an effort to develop empirical knowledge, as research on the psychosocial impact of the COVID-19 pandemic on Latinx young adults is limited. This method served to capture the richness of the experiences of participants by allowing analytic codes and categories to drive theory development. In total, seven focus groups were held and participants attended a virtual focus group with other Latinx emerging adults from their state. The focus groups were transcribed verbatim and coded using constructivist grounded theory. Five themes were identified from the data concerning the impact of the pandemic on Latinx emerging adults, which centered around mental health experiences, navigating family factors, pandemic-related communication, career and academic disruptions, and systemic and environmental factors. A theoretical model was constructed to generate an understanding about factors influencing psychosocial functioning for Latinx emerging adults during the pandemic. The study has implications for advancing science on the consequences of pandemics on mental health and cultural considerations that may influence disaster recovery. Examples of these cultural considerations that emerged from this study include multigenerational values, heightened responsibilities, and mediating pandemic information. Results can inform efforts to increase support and resources for Latinx emerging adults in order to address the psychological difficulties resulting from the COVID-19 pandemic.

## 1. Introduction

The COVID-19 pandemic has highlighted significant health disparities for Latinx and other ethnoracially minoritized groups in the U.S. Importantly, data show that Latinx have been overrepresented in COVID-19 cases and deaths [Center for Disease Control (CDC), 2022]. These disparities result from a broad range of contextual factors, such as the fact that during the shelter-in-place orders, a high proportion of Latinx were considered essential workers (e.g., farmworkers, custodians, and grocery clerks) and could not remain at home or work remotely, practice social distancing, or obtain the necessary personal protective equipment to reduce infection risk (Hayes-Bautista, 2020). Additionally, due to the high cost of living, many Latinx tend to live with more inhabitants per household and often reside in multigenerational homes, further increasing the probability of contracting COVID-19 (Hayes-Bautista, 2020). Other reasons include vulnerable Latinx, particularly those who are undocumented, having no health insurance, or being unable to seek attention from a medical doctor (Page et al., 2020). For example, many Latinx who are undocumented are afraid of being screened or seeking health-related services due to fear of repercussions, such as deportation (Page et al., 2020). The combination of these deeply rooted systemic injustices has augmented psychological distress for many Latinx in the U.S. and led to concerns regarding the consequences of the pandemic on the mental health of this ethnic group.

The COVID-19 pandemic has had a significant impact on the mental health of many Latinx communities in the U.S. (Czeisler et al., 2020; Breslau et al., 2021; Garcini et al., 2021). There are findings indicating that Latinx adults have experienced higher psychological distress during the COVID-19 pandemic than other ethnic groups (Czeisler et al., 2020; Garcini et al., 2021). One study found that Latinx reported higher symptoms of anxiety, depression, substance use than non-Latinx Whites or non-Latinx Asians during the pandemic (Czeisler et al., 2020). In addition, 10.7% of respondents in the study reported seriously considering suicide in the 30 days prior to the survey which was highest for ethnic/racial minorities and included Latinx (18.6%) (Czeisler et al., 2020). Longitudinal research has also found that Latinx were more likely to report an increase in psychological distress than other racial/ethnic groups (Breslau et al., 2021). Although there is research showing that the COVID-19 pandemic has impacted the mental health of Latinx, less is known about the mechanisms by which the impact of this pandemic has affected the mental health of this population.

### 1.1. Emerging adults and mental health during the COVID-19 pandemic

Emerging adulthood is a developmental stage of young people ages 18–29 (Arnett, 2015). This developmental stage is characterized as a time of: (1) *identity development*, which involves considering and trying various life options; (2) *instability*, which involves instability in work, love, and place of residence; (3) *self-focus*, which involves taking time to focus on directing one’s life; (4) feeling *in-between*, not quite an adolescent or an adult; and (5) experiencing a sense of *possibilities/optimism* regarding life opportunities (Arnett, 2015). How people experience emerging adulthood is likely to vary considerably across national, cultural, and socioeconomic contexts (Arnett, 2015). During the height of the COVID-19 pandemic, long-term vocational and social growth for emerging adults was narrowed through curtailed education, limited ability to travel, lack of opportunities to obtain vocational training, and limited employment opportunities (Gruber et al., 2020).

### 1.2. Latinx emerging adults and pandemic-related stressors

For Latinx emerging adults the pandemic posed unique challenges. These challenges include economic and social burdens, experiencing heightened communication inequities, living in multigenerational households with family members at high risk of contracting COVID-19, and isolation related to immigration-related factors (Villatoro et al., 2022). This is troubling considering Latinx emerging adults are the youngest major racial group in the U.S. and also play a vital role in contributing to the country’s future (Lopez et al., 2020). Hence, more research is needed to examine factors influencing their distress, protective factors, and adequate ways to promote their long-term adjustment.

Studies specifically examining the psychosocial impact of the COVID-19 pandemic on Latinx emerging adults have found that distress has increased for this group (Czeisler et al., 2020; Goodman et al., 2020; Penner et al., 2021; Villatoro et al., 2022). Patterns of elevated distress were shown to be persistent among Latinx emerging adults during the first 6 months of the pandemic (Riehm et al., 2020). This warrants attention as it has been well documented that Latinx emerging adults have lower rates of mental health service use than their White counterparts, and many experience barriers to treatment (Maani and Galea, 2020). Additionally, for Latinx emerging adults, mental health stigma has been identified as a unique predictor of help-seeking attitudes and is related to negative attitudes about treatment for this group (Mendoza et al., 2015; DeFreitas et al., 2018). Given the high prevalence rates of mental health symptoms in this population during the pandemic, it is necessary for mental health providers to deliver culturally sensitive interventions and increase engagement with mental health services (Castaño et al., 2007).

Various cultural constructs and contextual factors are relevant to understanding the mental health experiences of Latinx emerging adults during the COVID-19 pandemic and to the topic of this research study. For example, Latinx generally have a relational orientation that helps shape their identity, family life, community, social world, and collectivist orientation (Comas-Díaz, 2006). Many young Latinx appear to adhere to *familismo*, a cultural value where one’s family is expected to provide necessary emotional and instrumental social support (Stein et al., 2019). Importantly, *familismo* can increase a sense of obligation, which involves caring for one’s family and considering family when making decisions (Valdivieso-Mora et al., 2016). *Familismo* has been reported to impact emotional proximity, affective resonance, interpersonal involvement, and cohesiveness (Comas-Díaz, 2006).

Since Latinx culture tends to endorse *familismo*, this aspect may translate into behaviors such as accepting various family responsibilities. For low-income Latinx emerging adults, having family obligations, and lacking economic resources has been shown to influence their decision to attend college due to a need to work, support, or care for other family members (Sánchez et al., 2010). Given the COVID-19 pandemic, it is likely that Latinx emerging adults may have experienced increased family obligations and have increased their support to other family members. Examples include caring for younger siblings or ill relatives or needing to take other jobs to help support family due to economic difficulties. Hence, a more in-depth understanding the role of *familismo* and heightened responsibilities is needed to better understand how this cultural value is linked to mental health outcomes for Latinx emerging adults in the pandemic context.

Another consideration is that Latinx emerging adults tend to serve as language brokers or linguistic intermediaries for their families and relatives (Shen and Dennis, 2017). They may also assist in navigating across cultures and making important family decisions. For second-generation immigrants, language brokering usually starts between 8 and 10 years of age and has been associated with having higher levels of depression (Villanueva and Buriel, 2010; Rainey et al., 2014). Language brokers may sometimes report stress associated with this role, which can create a sense of burden and negatively influence family dynamics (Shen and Dennis, 2017). During the beginning stages of the COVID-19 pandemic, Latinx emerging adults may have provided information to their parents and relatives regarding safety measures and the pandemic due to the limited availability of information and culturally targeted public health messaging for the Latinx Spanish-speaking community.

For Latinx emerging adults, the developmental demands of emerging adulthood may be heightened by the additional stressors of being an ethnic minority within a majority culture and having to adjust to college or the workforce. Importantly, the prevalence of mental health diagnoses increases among second generation racial/ethnic minority immigrants with U.S.-born Latinx reporting higher rates of most psychiatric disorders than Latinx immigrants (Alegría et al., 2008; Salas-Wright et al., 2014). Other research has shown that being a second-generation immigrant was associated with increased perceived health risk from COVID-19 (Jamieson et al., 2021). With respect to the COVID-19 pandemic, Chavez et al. (2021) identified that Latinx young adults who were exposed to anti-immigrant rhetoric during the first year of the pandemic reported negative emotions including sadness and anger. Furthermore, Latinx emerging adults coming from immigrant families may be particularly vulnerable to experiences of social isolation due to discrimination, familial separation, and political climate-related factors which were heightened by the pandemic and led to compounded isolative effects (Hawkins et al., 2020).

Overall, *familismo*, family responsibilities, stigma, language brokering, and immigration factors are just a few of the factors that may impact the experience of Latinx emerging adults in the pandemic. They are important to consider in understanding variations in adjustment, identifying protective factors, and to develop culturally responsive pandemic-related interventions. Hence, an in-depth understanding of Latinx emerging adults during the COVID-19 pandemic and factors driving distress is imperative to better understand and respond to their specific needs, mitigate adverse educational outcomes, and adequately support their adjustment.

### 1.3. Present study

The current qualitative study aimed to explore the psychosocial impact of the COVID-19 pandemic on Latinx emerging adults (ages 18–29) through virtual focus groups conducted from December 2020 through April 2021. At the time when the study began research on the psychosocial impact of the COVID-19 pandemic was limited and most of the research studies in this area were quantitative and had a focus on clinical outcomes. Therefore, a qualitative constructivist grounded theory approach was used in an effort to develop empirical knowledge and to understand broadly the range of experiences of participants in an in-depth manner. The primary research question was the following: “What is the impact of the COVID-19 pandemic on the psychosocial functioning of Latinx emerging adults?”

## 2. Materials and methods

### 2.1. Research design

A constructivist grounded theory approach was selected as it served to capture the richness of the experiences and context of participants by allowing analytic codes and categories to drive theory development (Strauss and Corbin, 1990; Charmaz, 2014). In using this approach, knowledge was generated and interpreted through the interactions and lenses of the participants and the researcher (Haverkamp and Young, 2007). Additionally, the virtual focus group method was chosen for this study as it could foster a collectivist social environment for participants that could elicit exploration of beliefs and feelings associated with the COVID-19 pandemic (Liamputtong, 2011). Throughout all facets of the research process, the research team engaged in reflexivity and considered how their own social identities and positions affected the angle of investigation and interpretation of data. Prior to collecting data, each team member audio recorded a reflexivity statement and shared with the team their perspectives about the psychological impact of the pandemic on the Latinx community in the U.S. In addition, the research team discussed during weekly meetings how our social identities and personal factors influenced the research.

### 2.2. Participants and procedures

Institutional Review Board approval was obtained at the University of California, Santa Barbara. Recruitment began in November 2020 and continued through April 2021. The initial plan was to recruit participants who originally participated in a longitudinal, multi-site survey study regarding the psychosocial adjustment of young adults’ following devastating hurricanes and wildfires that occurred in 2017–2018. They were recent graduates and had agreed to be contacted again for future related studies. Those residing in California and Florida who self-identified as Latinx or Hispanic were invited to participate in the study *via* email and phone. Only eight participants were recruited from the longitudinal, multi-site survey study; therefore, the Institutional Review Board (IRB) protocol was modified to recruit other Latinx emerging adults in California and Florida. We continued with data collection from these two regions to be consistent with the existing sample and it provided the opportunity to obtain diversity in participants’ experiences during the pandemic given different state regulations in the pandemic. Although our aim was not to compare experiences between regions, we considered how varied settings, COVID-19 protocols, and context influenced Latinx emerging adults in California and Florida. The study was advertised on social media (Facebook and Instagram) and through word of mouth. Those interested in participating were screened and completed a brief phone briefing prior to being enrolled in the study. In order to meet criteria for the study participants had to identify as Latinx, reside in California and Florida, and be willing to partake in a focus group. In the consent form sent to each participant through a Qualtrics survey, the participant was informed about the study procedures. All participants were compensated for their participation with a $15 Amazon gift card.

A total of 35 Latinx individuals were recruited for this study but four participants who initially expressed interest were unable to be reached. Thus, the final sample consisted of 31 participants. By study design, participants were between the ages of 18–29 years (*M* = 23.32, SD = 2.73). In addition, 66.7% of participants were bilingual in English and Spanish. Over half of the participants (56.7%) reported that they had a family member that was considered an essential worker and a large percentage (83.3%) reported having family members that they considered to be at greater risk of contracting COVID-19. Over half (63.3%) were living in multigenerational homes at the time of the study. Additional information about the participant characteristics is displayed in Table 1.

**TABLE 1:** Demographic characteristics of study participants (*N* = 31).

Participants	*n*	%
Age range 18–29	31	100
**Gender**		
Female	25	80.6
Male	5	16.1
Non-binary	1	3.2
**Generational status in U.S.**		
1st generation	4	12.9
2nd generation	26	83.9
3rd generation	1	3.2
**Latinx descent**		
Mexico/Mexican American	19	61.3
Cuba/Cuban American	4	12.9
Puerto Rico/Puerto Rican	1	3.2
Other (Colombia, El Salvador, Honduras)	7	22.6
**Residence setting**		
Suburban	17	56.7
City/Urban	12	40
Rural	1	3.3
Enrolled as student	21	67.7
**University class**		
Freshman	1	3.3
Sophomore	3	10
Junior	4	13.3
Senior	9	30
Graduate student	5	16.7
Completed college	8	26.7
Currently working	24	80
**Income bracket**		
Less than $20,000	10	33.3
20,000–$49,999	8	26.6
50,000 or above	9	30
Preferred not to respond	3	10
Tested positive for COVID-19	6	19.4
Family member essential worker	17	54.8
Family member at high risk of contracting COVID-19	25	83.3

### 2.3. Measures

#### 2.3.1. Demographic form

Participants were asked demographic questions regarding their ethnic background, gender identity, primary language, education and/or work status, immigration generational status, income bracket, COVID-19 perceived risk and exposure (i.e., whether participants had COVID-19, if they had a family member or loved one who they considered to be at high risk for exposure to COVID-19, etc.), and residence setting (i.e., suburban, rural, or urban community). The demographic information was used to understand the context of each participant and to determine any key differences between the participants in each focus group.

#### 2.3.2. Interview protocol

A semi-structured interview protocol in English was developed, piloted, and revised. The researcher created 13 open-ended questions to address the research aims. The protocol was piloted by conducting a virtual focus group with three Latinx emerging adults in California who were similar to the target population. The pilot focus group allowed the researcher to obtain feedback about the nature of the study, to adjust any protocol questions, and identify themes that could emerge from participants.

#### 2.3.3. Memos

Memos of written notes about the data, behavioral observations, ideas, and assumptions were completed throughout the research process to adhere to a constructivist grounded theory approach (Charmaz, 2014). Memo-writing helped the research team to identify patterns, consider biases, and explore themes in the data.

### 2.4. Data collection

Seven focus groups were conducted for the study. All were conducted in English although there were certain words or sentences that participants discussed in Spanish. To adhere to COVID-19 social distancing protocols, and because of the geographical location of the participants, focus groups were conducted online using Zoom. The researcher and most participants had their cameras on during the focus groups. Participants were grouped based on geographical region (i.e., Florida and California). The focus group sessions ranged from three to seven participants and lasted approximately 1.5 h each. Focus groups were audio-recorded. After each focus group, participants were provided information regarding mental health resources and services. No participants reported concerns with regards to their privacy.

### 2.5. Data analysis

Focus groups were transcribed with software Otter.ai. which uses artificial intelligence and machine learning to transcribe audio. The transcript obtained from the software was then reviewed by a research assistant and the lead investigator with the focus group audio and edited to fix any errors and to remove any identifying information during the focus groups. Statements that participants made in Spanish were transcribed by the research team and translated by the lead researcher who is a native Spanish speaker. The transcript for each focus group was then used for data coding.

The research team analyzed the data using constructivist grounded theory methodology which involved comparing the responses of participants and identifying consistent themes that were emerging between participants and across the different focus groups. We examined similarities and differences to make analytic sense of the content and to gain more awareness of the experiences of participants. In addition, using this method allowed us to conduct explicit systematic checks, refine the analysis, and engaged in critical reflexivity (Charmaz, 2014). There were three stages involved in coding the data which included initial line-by-line coding, focused coding, and theoretical coding (Charmaz, 2014). The themes were consolidated, and the memos were reviewed. The theoretical categories were presented in an order that represented the experiences that appeared to be most salient and important to the lives of the participants and answered the research question. Example quotes representative of each theme and which highlighted the participant voices were also selected. Using constructivist grounded theory, data was gathered until each category was saturated, meaning that the research team no longer found that additional information was contributing to an understanding of the derived categories (Strauss and Corbin, 1990; Charmaz, 2014). After completing coding of seven focus groups, the research team agreed that saturation had been reached. At that point no new categories or leads were arising, and the categories were sufficiently dense. Upon reaching saturation, an external auditor reviewed the codebook and provided feedback on ways of clarifying definitions of the focused codes utilizing the constructivist grounded theory tenets.

## 3. Results

**FIGURE 1:**
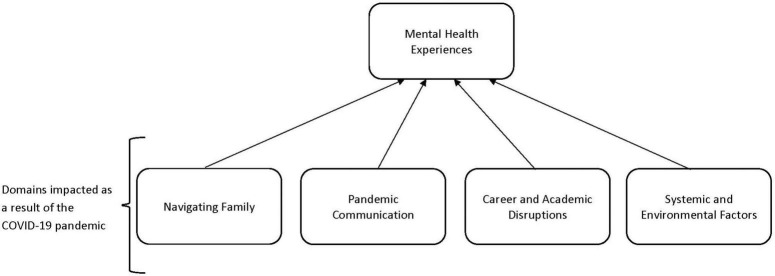
Model of psychosocial functioning of Latinx emerging adults as a result of the pandemic.

The data analysis resulted in a theoretical model regarding the psychosocial functioning of Latinx emerging adults during the COVID-19 pandemic. This model about psychosocial functioning included themes of psychological distress, reflections on mental health stigma, and a shifting mentality about self-care. The factors that were influential in terms of driving the mental health experiences of Latinx emerging adults included navigating family factors, pandemic communication, career and academic disruptions, and systemic and environmental factors. The thematic codes and sub codes that make up the model will be discussed below (see Table 2 for list of codes and Figure 1 below for model).

**TABLE 2:** Theoretical codes and related codes.

Theoretical codes	Related codes
Mental health experiences	1. Psychological distress
	2. Mental health stigma
	3. Mentality about self-care
Navigating family factors	1. *Familismo* and multigenerational values
	2. Heightened responsibilities
Pandemic communication	1. Mediating pandemic related communication
	2. Impact of media exposure
Career and academic disruptions	1. Transition to virtual format
	2. First generation experience
Systemic and environmental factors	1. Increased awareness of social disparities
	2. Barriers to accessing pandemic resources
	3. Pandemic climate

### 3.1. Mental health experiences

Participants reported experiencing significant interruptions in their daily lives which led to a range of mental health experiences. For many participants, the pandemic had taken a significant toll on their mental health and had generated distress. For those with pre-existing mental health disorders, the pandemic aggravated their symptoms or led them to seek additional mental health services. There were seven participants who voluntarily reported either having started to see a therapist or obtaining mental health services during the pandemic. There were many instances where participants described how mental health stigma in the Latinx community often made it hard for them to discuss their mental health experiences openly with their loved ones during the pandemic. In addition, when discussing mental health experiences, some reflected on how the pandemic had encouraged them to prioritize and value self-care as a way of coping with pandemic-related distress. The areas that pertained to the mental health experiences of participants are described below.

#### 3.1.1. Psychological distress

Collectively, participants described experiencing substantial psychological distress due to the COVID-19 pandemic, and for most, the distress was still present at the time when the study was conducted, which was between 13 and 17 months into the pandemic. Notably, participants reported experiencing depression, anxiety, irritability, withdrawal, difficulty sleeping, psychosomatic complaints, rumination, and a sense of helplessness or guilt. Below are two accounts of participants, one in Florida and one in California, that described their emotional distress during the beginning of the pandemic:

I had feelings of impending doom, there were days, when I would probably cry a lot. There were other days when I felt better, and then I would go back to the crying or to the anxiousness. (Participant 3, Age 24, Woman, Florida)

I think my mental health has definitely been impacted a lot. I’ve been getting so anxious. I’ve not gotten depressed, but I’ve had a lot of symptoms of depression. (Participant 1, Age 23, Woman, California)

Various participants shared that although they did not have a specific diagnosis for a mental health disorder or were not receiving mental health services, they were noticing mood changes, difficulty sleeping and concentrating, and a decrease in motivation. A participant shared the following:

I didn’t have the motivation to really do much because I didn’t really feel very well. So that really took a huge toll on my mental health. (Participant 12, Age 23, Woman, California)

In general, participants reported augmented distress due to undergoing the pandemic and this distress was impacting their functioning with regards to engaging in activities of daily living, socialization, and academic engagement. Given, their psychological distress some had decided to seek mental health services and were concerned about their psychological wellbeing.

#### 3.1.2. Mental health stigma

The topic of mental health stigma emerged consistently throughout the focus group interviews as it related to having a Latinx identity. For example, some participants discussed their perceptions of how Latinx culture has a high level of stigma about mental health problems, and for them the stigma had become more apparent during the pandemic. Notably, a participant in California described being unable to communicate with family about experiencing mental health symptoms because they did not feel comfortable sharing or having a conversation about mental health.

I’m not transparent and I don’t communicate about my mental health, I’ve been more distant because the moment that I reach out to my mom, it’s like, hola como estas? (hi how are you?) And I have to lie every time. (Participant 20, Age 28, Woman, California)

When talking about stigma in the Latinx community participants exhibited frustration and reported wishing that mental health stigma would decrease as they felt that it impacted them and their community. Notably, some participants felt hopeful that the pandemic could promote the importance of mental health for the Latinx population.

#### 3.1.3. Mentality about self-care

One dimension of coping that emerged when discussing mental health experiences with participants was self-care. Importantly, participants expressed that due to the pandemic they had shifted their view about self-care and were making it a higher priority to promote their wellbeing and reduce stress due to changes in their routine. According to participants, before the pandemic, self-care was not a priority or was considered a luxury by their families, yet they felt that this had shifted and was becoming a more important aspect for coping during the COVID-19 era. For participants, self-care looked differently based on each participant; however, some provided examples of how they were taking time to engage in self-care activities such as painting, reading, exercising, baking, listening/creating podcasts, going into nature, getting sunlight, spending time with pets, journaling, watching television shows, and connecting with friends. Some others also reflected that they were practicing self-care by putting less pressure on themselves.

Before… COVID a lot of people didn’t really do (self-care) that or didn’t really think it was a priority. But I found that it’s very valuable to take that time for yourself. (Participant 12, Age 23, Woman, California)

Other participants discussed how the pandemic had motivated them to prioritize their mental health and to identify their wellbeing as their own responsibility.

I think balance is important. I think that’s something that the pandemic taught me as, as a young person and Latin person, balance between family, work life, friendships, caring for yourself… Your mental health and your physical health is definitely more important than anything at all. I wasn’t able to work when I had COVID, I wasn’t able to do anything because I had fatigue, and I had a lot of bad feelings in my body… I guess it was a sign that I wasn’t taking care of myself well enough and that I was kind of taking my youth for granted. (Participant 31, Age 24, Woman, Florida)

#### 3.1.4. Summary of mental health experiences

Overall, participants reported experiencing a range of symptoms of distress including depression, anxiety, difficulty sleeping, hopelessness, somatization. For some participants, the pandemic had shifted their sense of motivation. Participants consistently expressed the challenges associated with mental health stigma and reported finding it challenging to talk to their families and relatives about their distress. In addition, others described that the pandemic had served as an opportunity to prioritize self-care.

### 3.2. Navigating family factors

In the context of the pandemic, navigating family factors was a theme that emerged as influencing the psychosocial functioning of the participants. Helping family manage stressors such as identifying COVID-19 related resources or managing financial concerns brought about additional stressors during the pandemic, but as noted by participants it also served as a protective factor. The related themes under this code included *familismo* and multigenerational values, as well as heightened responsibilities.

#### 3.2.1. Familismo and multigenerational values

Most participants discussed adhering to *familismo*. For example, one participant described being highly concerned about the wellbeing of their family and how this impacted them:

But for my parents it’s a lot different and I think as a Latinx young adult, their wellbeing falls on my wellbeing as well, like, I’m not okay, if they’re not okay. (Participant 20, Age 28, Woman, California)

Another participant who moved out of her family’s home during the pandemic reported having difficulty entertaining herself alone and expressed appreciation for living in a multigenerational household.

But now that I am not with my family, it is a little more difficult trying to find ways to entertain myself alone, not having that multigenerational household where I always had the social aspect whenever I wanted. (Participant 4, Age 24, Woman, California)

Another participant described experiencing a mixture of emotions regarding family during the pandemic. They stated:

I have always been worried I’m going to cause them to contract COVID but at the same time, having all that support in one household, there is always someone you can speak to or just think about things with them. There’s not really a quiet moment. You always have someone to speak to you when you’re not allowed to go out, you’re not really supposed to be speaking to so many people you have this small circle of a small family, smaller community inside your own house (Participant 4, Age 24, Woman, California).

In general, living in a multigenerational home was described as challenging for many participants, both in terms of protecting their relatives from COVID-19 and juggling additional family responsibilities. On the other hand, the pandemic had also made it easier to get emotional support by increasing opportunities to share time with relatives and to promote a sense of community at home when so many were isolated.

#### 3.2.2. Heightened responsibilities

Participants reflected about having increased family obligations during the pandemic, including helping around the house, taking care of family members, and making financial contributions. Participants shared that their responsibilities had increased during COVID-19. This was often due to their parents being essential workers, needing more support, being older, or at a higher risk of contracting COVID-19. Below is an example of a participant discussing their perceptions of taking on responsibilities.

I am the youngest, so I was always like going out and getting groceries and kind of taking that responsibility to protect everyone else (Participant 30, Age 26, Woman, Florida).

#### 3.2.3. Summary of navigating family factors

Participants reported concerns about the safety and wellbeing of their family during the COVID-19 pandemic and experiencing high levels of stress due to potentially exposing family to COVID-19. Participants described that living back home posed challenges with regards to having to take on additional household responsibilities, caring for other relatives, encountering increased academic hardship as a result of family experiencing unemployment, and encountering limited space to work and study. Nonetheless, participants shared a deep sense of gratitude for their families and were appreciative of the wisdom of older relatives during the pandemic. Furthermore, participants reported that the pandemic had provided an opportunity for them to practice setting more boundaries with family in order to prioritize work and academics during that time.

### 3.3. Pandemic communication

Throughout the focus groups, participants reflected about their experiences serving as language or information brokers to their families for the following reasons: their parents or older relatives did not speak English and lacked accurate public health information in Spanish, they had difficulty understanding COVID-19 safety protocols, or needed advocacy. Participants in California and Florida described that in the process of providing information and interpreting public health or safety protocols, they felt a sense of duty or pressure to present accurate and trustworthy information to keep their loved ones safe. Additionally, many indicated that they spent time identifying accurate public health information to support their families in advocating for their safety at work because many of them were essential workers and had little to no flexibility when it came to working remotely. Some also discussed the impact of prolonged and repeated exposure to media and graphic pandemic-related images. There were two related themes that emerged: mediating pandemic-related information and impact of media exposure.

#### 3.3.1. Mediating pandemic related information

Participants described the way they supported their families even prior to the pandemic by navigating across cultures and often served as language brokers to relatives who did not speak English, but this increased significantly since the implementation of COVID-19 safety and health protocols. Participants often had to provide information regarding safety measures to their parents and relatives due to limited information available in Spanish or inaccurate information that their family was obtaining from Spanish news channels regarding COVID protocols. Participants expressed that they felt a responsibility to deliver pandemic-related information accurately and were often spending time educating relatives about COVID-19 or making difficult decisions about the safety of their parents and elders due to pandemic-related protocols. A participant shared:

The pressures to deliver the right information and trying to figure out the sources that are actually saying the right things. (Participant 3, Age 20, Woman, California)

From the viewpoint of participants, information on Spanish news channels was often sensational or inaccurate. One participant discussed how the information that they were obtaining heightened their distress and created additional confusion.

I feel like even the regular news… sometimes the wording is not necessarily great. And then the things that people will talk about in the Hispanic community, there was a lot of people very scared, and a lot of misinformation being spread around. (Participant 30, Age 26, Woman, Florida)

According to participants, mediating pandemic-related information was a significant factor contributing to emotions of burden and fear, particularly during the initial part of the pandemic. Some also expressed disillusionment by communication inequities in public health messaging and how this was impacting predominately Spanish speaking Latinx.

#### 3.3.2. Impact of pandemic media coverage

Participants described feeling overwhelmed and distressed by the media coverage that they encountered during the initial phases of the pandemic. Various participants felt conflicted, as they wanted to remain informed so they could protect themselves and their families, but were overwhelmed by the heightened emotional reactions and the distressful content that they were being exposed to relating to social inequities and how the pandemic was impacting ethnic minorities. Furthermore, several participants discussed how they were not just overwhelmed by the media content that they were obtaining regarding their state or community, but also felt concerned about the news that they were receiving regarding the impact that the pandemic was having overall in Latin America.

It’s just been too much, like, all that’s on my plate plus, like everything with COVID or like the news and worrying about my family, the family here, the family in Mexico, and all that was just stress that I did not have before the pandemic (Participant 16, Age 21, Woman, Florida).

#### 3.3.3. Summary of pandemic communication

Participants shared extensively about feeling some pressure to inform their families about taking safety precautions during the COVID-19 pandemic. Some were also frustrated by inaccurate information being shown on Spanish news channels. In addition, participants reflected on the psychological impact of having constant exposure to news and updates regarding the pandemic and how their stress was compounded by the pandemic-related information shifting so rapidly.

### 3.4. Career and academic disruptions

Most participants described how the COVID-19 pandemic had led to drastic disruptions in their academics or employment and had brought about many challenges for Latinx emerging adults. The primary themes related to this topic were: transitioning to a virtual format, loss of opportunities, change in career plan, and first-generation experience.

#### 3.4.1. Transitioning to virtual format

As a result of the safety precautions put in place to prevent the spread of COVID-19, participants shared their experiences of the psychological impact of transitioning to virtual learning or working. Several participants mentioned that studying and working virtually had affected their mood, motivation, sleep, and productivity. In addition, due to their home environments and the burdens brought about by the pandemic, participants reported that it was difficult to stay focused on academics and work. They also described having trouble connecting and communicating with faculty and colleagues virtually, leading to feelings of isolation, low motivation, and diminished effectiveness. Generally, participants attributed their difficulties in keeping up with academic work due to a lack of academic support, managing family expectations, and lacking a distraction-free environment in their home to complete their work or attend virtual classes. One participant shared the following:

I abruptly transitioned to work from home, the work wasn’t the same, I wasn’t feeling fulfilled. And then I would close my laptop, but then it’s like, what do I do now? Right? my life was just between four walls for an undetermined amount of time. So, I had little bouts of, I guess you can say depression, but it was really just more like low moods, low affect not really motivated to do much (Participant 29, Age 26, Woman, Florida).

Participants reported that they struggled so much academically in virtual classes that they had to adjust their expectations about their performance, shift their career plans, or take time off from studying. For example, a participant stated the following:

Latinx students in our university might not have the resources to have a stable internet connection or have that economic possibility to get a tutor or other outside resources that you need to continue to be successful in college. There are other struggles and other things that they’re going through than a White student might not be going through (Participant 3, Age 20, Woman, California).

#### 3.4.2. First generation student experience

Although we did not explicitly ask participants about their parents’ level of education. Many participants shared during the focus groups about the challenges that they encountered as first-generation students during the pandemic. They reported having limited knowledge with regards to navigating their academics or lacking support and guidance. For these students, having campus resources was crucial and returning home due to the pandemic made it difficult for them to concentrate, to feel a sense of mastery in their coursework, or to ask career-related questions. A first-generation student shared the following:

I don’t know what I’m doing or where I’m going. And I didn’t have the opportunity to have parents who went to college, as well. So, I don’t really have anyone to help me out, you know? (Participant 12, Age 23, Woman, California).

#### 3.4.3. Summary of career and academic disruptions

Participants described how academic and work-related disruptions were significantly impacting them. There was a shared concern regarding disrupted income and participants discussed feeling less engaged in academics and less hopeful about their work prospects. They reported difficulties focusing on work due to lack of space at home or the distraction of taking on additional responsibilities. With regards to these disruptions brought about by the pandemic, participants reported a sense of loss from a lack of in-person interactions with peers and coworkers, missing the opportunity to live on a college campus, and felt worried about the uncertainty of their future academic and career prospects.

### 3.5. Systemic and environmental factors

The theme of systemic and environmental factors reflects experiences participants shared about encountering systemic disparities during the pandemic and the environmental factors that influenced their pandemic experiences and psychosocial functioning. Three related themes were identified: increased awareness of social disparities, barriers to accessing pandemic resources, and the pandemic climate. Participants reflected on the challenges that they encountered navigating service systems (e.g., healthcare, immigration resources) to support their families during the pandemic. Furthermore, participants reflected on how the political polarization increased hostility and made it sometimes challenging to engage in conversations with others due to different perspectives and stances on the COVID-19 safety protocols and race related issues.

#### 3.5.1. Increased awareness of social disparities

Participants indicated that although they had previously experienced issues of discrimination and racial injustice, their awareness of systemic inequities and how they impact Latinx in the U.S. had become more visible as a result of experiencing COVID-19. They described how ethnic minorities had been disproportionately affected by the pandemic and they had gained a deeper understanding of how Latinx, undocumented immigrants, and those of indigenous background were at a disadvantage when it came to accessing COVID-19 testing, healthcare, and COVID-19 economic relief support. For example, a participant discussed noticing that many of the essential workers were Latinx.

And at the COVID testing centers here almost every worker that you see is Latinx. And that’s something I’ve noticed from the beginning of the pandemic, that just bothers me so much. Because why, you know, like, in the residence halls too the workers, they’re the ones that are cleaning our bathrooms in our rooms, like they’re all Latinx and they’re the ones at the bottom of this totem pole. And it’s so frustrating because I don’t know what I can do about it (Participant 25, Age 24, Male, California).

In addition, participants highlighted that they were more aware of disparities in resources they had at their disposal as Latinx young adults. Although not stated in this quote for some participants, gaining awareness and experiencing these differences in privilege made them feel overwhelmed and unmotivated. The participant shared:

Like the other communities that had more opportunities and more resources vs. like us, I feel like there’s that big racial disparity in what was available to us (Participant 28, Age 21, Female, California).

#### 3.5.2. Barriers to accessing pandemic resources

The focus groups revealed that participants had difficulties locating resources related to pandemics, including COVID vaccine testing, as well as barriers to accessing mental health care. Participants reported that encountering these barriers added additional stress to their lives and confirmed their perception of social inequities due to ethnicity, immigration status, and income level. In one case, a participant in the study shared the challenges she experienced in trying to obtain healthcare for her mother who became extremely ill with COVID-19. The participant described having to go to a different hospital outside of their residential community to access adequate healthcare, as their nearby hospital had turned them away. The participant stated:

She (mother) was very, very sick, she was hospitalized, and she was put on a ventilator. And I think for me, just how this experience was unique, maybe to someone who what isn’t from a Latinx background, is just that access to adequate health care. You know, being from a low-income background, growing up in a very low-income community, predominately Hispanic community. We’re surrounded by hospitals that aren’t the best… and so, we had to go out of our way and drive very far away to go to a better hospital that had better resources (Participant 1, Age 23, Woman, California).

#### 3.5.3. Pandemic climate

Participants discussed how the distress that they were experiencing was not limited to COVID-19, but also to the political climate and navigating perspectives regarding pandemic-related safety protocols with others. Participants discussed how political views were impacting perceptions of COVID-19 safety precautions, which created additional stress and frustration. A participant shared:

It really is difficult if you’re younger, and you have a political view that is so different from the majority of other people. You try not to see it from a political point of view, you understand it’s a virus and you have to take care of yourself and take care of other people (Participant 30, Age 26, Woman, Florida).

#### 3.5.4. Summary of systemic and environmental factors

Participants became more aware of racial health disparities and systemic inequities due to experiencing the pandemic and this exposure contributed to their distress. They also discussed the barriers that the Latinx community faced during the pandemic and provided examples of how these inequities affected them personally. Another source of stress that emerged was related to the political divide in the U.S. and the ways in which the pandemic climate influenced their perspectives on safety protocols.

## 4. Discussion

The COVID-19 pandemic has disproportionately affected communities of color in the U.S. and has exposed racial and systemic injustices that can put at risk the physical and emotional wellbeing of Latinx (Garcini et al., 2021). The present study utilized a constructivist grounded theory approach to explore the psychosocial functioning of Latinx emerging adults during the COVID-19 pandemic. The aim was to explore the psychosocial impact of the COVID-19 pandemic on Latinx emerging adults and to identify factors influencing mental health outcomes for this group. The themes that emerged from the data are valuable in clarifying aspects driving distress for Latinx emerging adults in the pandemic. The theoretical model of psychosocial functioning that emerged illustrates the complexity of factors associated with Latinx psychosocial functioning. The model outlined the domains of navigating family, pandemic communication, career, and academic disruptions, as well as systemic and environmental factors that influences the mental health experiences of participants during the COVID-19 pandemic. In addition, the model recognizes that Latinx emerging adults are interacting with their environment dynamically, which serves to inform how they have adapted based on their mental health experiences, social relationships, and resources. Furthermore, the model draws attention to areas (i.e., family, academics) where this population may need additional support in a pandemic context.

The study findings serve to alert the public’s attention about Latinx emerging adults experiencing compounding challenges in the pandemic that contributed to them experiencing a range of psychological symptoms (e.g., anxiety and depression). The psychological distress reported in this sample reflect emerging research showing that overall, young adults have experienced an alarming increase in adverse mental health outcomes and suicidality result from the COVID-19 pandemic (Liu et al., 2020; Conrad et al., 2021). For example, Center for Disease Control survey data collected during the summer of 2020 showed that those between the ages 18–24 had higher rates of anxiety, depression, trauma, and suicidality than any other age cohort (Czeisler et al., 2020). In terms of Latinx emerging adults, studies have found that this population has experienced clinical levels of mental health symptoms during the pandemic (Goodman et al., 2020; Villatoro et al., 2022). For the participants in our study, the distress they experienced impacted their work performance, presented challenges to their family lives, influenced their socialization, and shifted their perspective of the future.

Mental health stigma was important to participants as it made it challenging for them to communicate openly about their emotions and adjustment during the pandemic. Prior research has shown that older generations of Latinx may report higher rates of shame and embarrassment about having a mental illness than non-Latinx Whites, thus limiting their help-seeking for mental health problems (Jimenez et al., 2013). Additionally, Latinx parents and their children are less likely to agree about the severity of mental health problems, with findings suggesting that Latinx parents may only observe distress associated with more severe symptoms (Roberts, 2005). Stigma may serve as a barrier to obtaining services for Latinx children significantly more than non-Latinx White parents (Chavira et al., 2017). The reports of psychological distress and perceived mental health stigma among current study participants, highlight the need to improve the mental health literacy of family members, as this may play a role in how they can support the mental health of Latinx emerging adults.

Participants reported that family dynamics contributed to their stress during the pandemic. This stress was driven by the fear of having family members contract COVID-19 due to their work context or through close contact with relatives. Participants often reported feeling overwhelmed by seeking pandemic relief resources for family and encountering additional responsibilities at home. Research has extensively documented that Latinx culture tends to endorse *familismo*, which encompasses both value endorsement (i.e., attitudinal familism) and enactment of behaviors (i.e., behavioral familism) (Stein et al., 2019). Hence, *familismo* may translate into behaviors such as the provision of support to family members and taking on a variety of family responsibilities (Stein et al., 2019). Study results showed that *familismo* as a value played an important role in the mental health of participants during the pandemic and promoted additional worry for the participants as they navigated additional family responsibilities. For these Latinx emerging adults, the pandemic brought about unique challenges regarding protecting and advocating for family.

Notably, spending quality time with parents and relatives also appeared to be protective in decreasing isolation and promoting a sense of social support for participants. Perceived support has been consistently positively associated with better post-disaster adjustment among adults and children (Bonanno et al., 2005; Kaniasty, 2020). Additionally, studies have specifically linked perceived social support with a resilient mental health outcome after a disaster (Bonanno et al., 2008). In this study, it appeared that many participants felt supported and cared for, yet there were also areas, particularly with regards to speaking about their emotions or mental health during the pandemic, where participants felt less supported by their family and relatives. Accordingly, it may be important to consider that family factors can create additional distress, while at the same time enhance perceptions of social support.

Latinx emerging adults reported being psychologically affected by pandemic-related media exposure. Specifically, participants shared feeling overwhelmed due to being trusted informants to their families and having the responsibility of passing along COVID-19 safety information to parents and relatives who they felt may not be able to obtain up-to-date or trustworthy pandemic-related information in Spanish. Aligned with these findings, a study with Latinx from Laredo, Texas, found that children often serve as information brokers for parents and relatives participating as opinion leaders (Soto-Vásquez et al., 2020). Overall, these findings shed light on the role that Latinx youth play in keeping their families informed about public health safety protocols. The findings also highlight the need for accurate reporting of science in media in multiple languages and provides insight regarding the quality of public health content that Latinx in the U.S. may encounter in a disaster context. In addition, participants reported being impacted by exposure to pandemic-related news *via* multiple outlets (e.g., television, social media, live footage, and internet blogs). Prior research has shown that repeated media exposure to community crises may impact physical and psychological health (Pfefferbaum and North, 2020; Silver et al., 2021). With the intense media coverage of tragedies and disasters, it is essential to further understand the mechanisms by which diverse emerging adults cope with media disaster content.

Latinx emerging adults’ academic and professional paths were disrupted by the pandemic. Specifically, the study found that remote learning and working transitions and loss of face-to-face encounters negatively impacted the ability of participants to cope with academic and work demands. Some of the aspects that made the pivot to virtual learning and working most challenging for participants involved having limited space in the home to focus on online learning, time demands, and working as well as lacking social interactions with peers and colleagues. These findings are well supported by multiple reports that have addressed concerns regarding the remote learning transition, technology barriers, and perceived lack of university support during the COVID-19 pandemic (Hasan and Bao, 2020; Cortés-García et al., 2021; Halliburton et al., 2021). Notably, the results from this study indicate that Latinx emerging adults may need additional support with academics and job transitions due to some of the barriers and difficulties that they experienced adjusting to virtual formats and working more independently during the initial stages of the pandemic. Additionally, the study highlights that Latinx emerging adults who are first-generation students may need extra academic support and mentorship. The pandemic has shifted learning environments and therefore developing sustainable interventions to support first-generation students could reduce barriers in academic settings and promote their academic success.

Results demonstrate the significant impact that systemic, social determinants of health, and racism have with respect to impacting the wellbeing of Latinx emerging adults during the pandemic. The mental and physical health impact of exposure to social inequities has been well documented for people of color (Shi et al., 2003; Alegría et al., 2008). The findings of the study provide insight into the social, political, economic, and environmental stresses faced by the Latinx community, as well as how disparities and injustices have been magnified by the COVID-19 pandemic. The current study’s results are consistent with a recent qualitative study found that Latinx college students reported were worried about the future due to encountering social injustices during the pandemic (Morgan and Zetzer, 2022). In our study, several participants reported experiencing barriers to accessing resources for themselves and their families, including the inability to obtain COVID-19 testing and health care, which led to additional burden and distress. The findings highlight the need to implement additional resources for communities of color. Specifically, as it related to addressing inequities across sectors by improving stable housing, unemployment benefits, food security, healthcare, and mental health services.

## 5. Strengths

This study makes a significant contribution to the literature by using qualitative methodology to examine the psychological impact of the COVID-19 pandemic on Latinx emerging adults. This method provided nuance regarding the perceptions of the pandemic from participants, it generated new insight about cultural factors that may mediate disaster adjustment and psychosocial functioning for this population experiencing barriers to pandemic-related resources, heightened responsibilities, and having to relay disaster information. The study also examined Latinx young adults from different regions of the U.S. and integrated the experiences of Latinx young adults with diverse descents, varied levels of education, and socioeconomic status. One of the benefits of conducting a focus group study was that it allowed for a rich discussion and for participants to recall information that they may not have shared or remembered if they had participated in individual interviews.

## 6. Limitations and future direction

The study is not without limitations that should be taken into consideration. As a result of the focus group online format, it is possible that some participants may have felt uncomfortable discussing information with the group and may have had privacy constraints in their homes. In addition, it should be noted that some participants were more talkative than others, and as a result, some of them may not have shared in depth about their personal experiences. The study did not inquire specifically about how participants arrived at their identity status or what it meant for them to be Latinx. It is possible that the category of Latinx may not fit a participant as well, and there may have different levels of acculturation that were not captured in the study. Future research should include questions/measures that can capture identity aspects and examine generation of immigration among emerging adults. Finally, the mental health experiences recalled by participants could have been different had the study taken place during the early stages of the pandemic when lockdowns were in effect and less information was available about COVID-19. Future research utilizing multi-method approaches with a larger sample would further enhance understanding of the impact of COVID-19 pandemic on this population. In addition, more research is needed on how Latinx emerging adults coped during the pandemic and informing on positive and maladaptive coping strategies. Understanding the factors affecting adaptation in their context will help in providing effective, culturally tailored resources and services.

Despite these limitations, the study adds important information about the mental health experiences of Latinx emerging adults. This is valuable as it provides a deeper level of understanding of pandemic-related factors influencing distress, their social resources, and areas for consideration for protecting the mental health of this population. Notably, the results clarify the various areas where young adults may need additional support and underlines protective factors.

## 7. Study implications

Overall, this study demonstrates that it is imperative that clinicians, counselors, education professionals, and family members recognize the various factors and systems which have psychologically impacted Latinx emerging adults during the COVID-19 pandemic. Mental health providers and those working with Latinx emerging adults should consider the importance of social determinants of health (e.g., regional communities, economic stability, acculturation level, social resources) that can influence the experience of Latinx emerging adults and exacerbate stress for this population. Based on our findings regarding the role of communication, more culturally targeted COVID-19 messaging in Spanish may reduce the burden placed on Latinx emerging adults to serve as information brokers. For example, having Spanish translation of COVID-19 press conferences by government leaders could be useful with regards to reducing pandemic-related misinformation. The study findings also highlighted the role of family relationships for Latinx. Hence, if some of the stressors affecting Latinx families during the pandemic are reduced (i.e., limited caregiving, barriers to health care, and increased targeted public health information in Spanish), then it is likely that the mental health of Latinx will also be improved (Garcini et al., 2021). Based on the accounts of participants with regards to their exposure to social injustices, it is important for mental health providers to address the psychological impact that social injustices have had on them during the pandemic and to promote sources of strength and meaning (Morgan and Zetzer, 2022).

Based on the findings of the study with regards to academic disruptions, university staff should implement interventions to enhance the wellbeing of first generation Latinx students who have returned to college campuses or completing degrees remotely. Universities can work with administrators, faculty, and staff to adjust evaluation systems, promote flexibility in workload expectations, and provide alternative pathways to promoting career goals (Wolff et al., 2020). In addition, universities should offer additional opportunities for first generation students to engage in campus activities, build social support with peers, and obtain career-related mentorship. Universities can also collaborate with service offices to connect Latinx emerging adults to community/campus resources that promote instrumental support such as housing, food security, and financial aid. Lastly, future research is needed to continue to inform on the mental health of Latinx emerging adults in the long-term context of the COVID-19 and to identify areas to promote resilience and wellbeing as this population navigates the changing landscape and long-term impact of the COVID-19 pandemic.

## 8. Conclusion

This exploratory study identified navigating family, pandemic communication, career and academic disruptions, and systemic and environmental as influential factors impacting the mental health experiences of Latinx emerging adults during the COVID-19 pandemic. Our hope is that the current study is one step forward in continuing to improve our knowledge about factors driving distress for Latinx in the context of the COVID-19 pandemic and that study findings provide insight regarding ways to increase mental health, academic and career prospects, and adjustment for this population.

## Data availability statement

The raw data supporting the conclusions of this article will be made available by the authors, without undue reservation.

## Ethics statement

The studies involving human participants were reviewed and approved by the Institutional Review Board at University of California, Santa Barbara. The patients/participants provided their written informed consent to participate in this study.

## Author contributions

Both authors are responsible for the design and conception of the study, writing the drafts, and revising the manuscript.
